# Improvement on the productivity of continuous tequila fermentation by *Saccharomyces cerevisiae* of *Agave tequilana* juice with supplementation of yeast extract and aeration

**DOI:** 10.1186/s13568-016-0218-8

**Published:** 2016-07-22

**Authors:** Guillermo Hernández-Cortés, Juan Octavio Valle-Rodríguez, Enrique J. Herrera-López, Dulce María Díaz-Montaño, Yolanda González-García, Héctor B. Escalona-Buendía, Jesús Córdova

**Affiliations:** Área de Biotecnología Industrial, CIATEJ Centro de Investigación y Asistencia en Tecnología y Diseño del Estado de Jalisco, Av. Normalistas 800, Col. Colinas de la Normal, 44270 Guadalajara, JAL Mexico; Systems and Synthetic Biology Group, Department of Biology and Biological Engineering, Chalmers University of Technology, Kemivägen 10, SE-412 96 Göteborg, Sweden; Department of Wood, Cellulose and Paper, Universidad de Guadalajara, Km. 15.5 Carretera 9 Guadalajara-Nogales, 45010 Zapopan, JAL Mexico; Department of Biotechnology, Universidad Autónoma Metropolitana Iztapalapa, Mexico City, Mexico; Department of Chemistry, Universidad de Guadalajara, Blvd. Marcelino García Barragán 1421, 44430 Guadalajara, JAL Mexico

**Keywords:** Tequila, Continuous fermentation, Yeast extract

## Abstract

Agave (*Agave tequilana* Weber var. azul) fermentations are traditionally carried out employing batch systems in the process of tequila manufacturing; nevertheless, continuous cultures could be an attractive technological alternative to increase productivity and efficiency of sugar to ethanol conversion. However, agave juice (used as a culture medium) has nutritional deficiencies that limit the implementation of yeast continuous fermentations, resulting in high residual sugars and low fermentative rates. In this work, fermentations of agave juice using *Saccharomyces cerevisiae* were put into operation to prove the necessity of supplementing yeast extract, in order to alleviate nutritional deficiencies of agave juice. Furthermore, continuous fermentations were performed at two different aeration flow rates, and feeding sterilized and non-sterilized media. The obtained fermented musts were subsequently distilled to obtain tequila and the preference level was compared against two commercial tequilas, according to a sensorial analysis. The supplementation of agave juice with air and yeast extract augmented the fermentative capacity of *S. cerevisiae* S1 and the ethanol productivities, compared to those continuous fermentations non supplemented. In fact, aeration improved ethanol production from 37 to 40 g L^−1^, reducing sugars consumption from 73 to 88 g L^−1^ and ethanol productivity from 3.0 to 3.2 g (Lh)^−1^, for non-aerated and aerated (at 0.02 vvm) cultures, respectively. Supplementation of yeast extract allowed an increase in specific growth rate and dilution rates (0.12 h^−1^, compared to 0.08 h^−1^ of non-supplemented cultures), ethanol production (47 g L^−1^), reducing sugars consumption (93 g L^−1^) and ethanol productivity [5.6 g (Lh)^−1^] were reached. Additionally, the effect of feeding sterilized or non-sterilized medium to the continuous cultures was compared, finding no significant differences between both types of cultures. The overall effect of adding yeast extract and air to the continuous fermentations resulted in 88 % increase in ethanol productivity. For all cultures, pH was not controlled, reaching low pH values (from 2.6 to 3). This feature suggested a reduced probability of contamination for prolonged continuous cultures and explained why no significant differences were found between continuous cultures fed with sterilized or non-sterilized media. Concentrations of volatile compounds quantified in the distillates (tequila) were in the allowed ranges established by the Mexican regulation of tequila (NOM-006-SCFI-2012, Norma Oficial Mexicana: Bebidas alcohólicas-Tequila-specificaciones, 2012). The preference level of the distillates was similar to that of two well-known commercial tequilas. The results suggested the possibility of implementing this innovative technology on an industrial scale, attaining high productivities and using non-sterilized agave juice.

## Introduction

Agave plants are used in Mexico for the production of different alcoholic beverages; of which, tequila is the most popular. Tequila is produced by distilling fermented agave juice (*Agave tequilana* Weber var. azul). The tequila market has had a tremendous expansion (from 104 to 253 million of liters) from 1995 to 2012 (Tequila 2012); however, agave plants diseases, high production costs and elevated volumes of vinasses have threatened the sustainability of this industry; for that reason, tequila producers have searched biotechnological and organizational alternatives, for increasing the productivity and efficiency of their processes (Casas 2006).

During the tequila fermentation, sugars from agave juice are converted mainly into ethanol, biomass and low amounts of aroma metabolites, which are responsible for the product sensory qualities. Due to its simplicity, batch cultures are traditionally used for tequila fermentation, which is generally recognized as the limiting step of the whole tequila manufacturing process, because of the long time period required (up to 7 days, for spontaneous fermentations) (Cedeño 1995). Compared to batch fermentations, continuous cultures have higher productivities and lower labor costs (Boulton and Quain 2007; Jackson 2008). Since the 1950s, some attempts have been made for beer and wine production using continuous fermentations. Although, this technological alternative might bring attractive advantages to the tequila process, few continuous fermentations of agave juice have been performed and most of them have been regarded as an analytical tool, implemented to characterize physiologically yeasts isolated from spontaneous tequila fermentations, rather than a production alternative (Díaz-Montaño 2004; León Murillo 2005). Only three studies were focused on the utilization of continuous fermentations for tequila production purposes. (1) Del Valle-Ruiz (del Valle Ruíz 2007) reported a pilot scale continuous culture of *S. cerevisiae* fed with agave juice; however, the productivity found in this system was lower than that reached in batch fermentation. This result might be explained by a microbial contamination or a nutrimental deficiency in the agave juice. (2) Moran-Marroquín et al. (2008) studied the fermentative capacity and synthesis of aroma compounds of *S. cerevisiae* at laboratory scale using different dilution and aeration rates, and supplementing ammonium phosphate in the fed agave juice. The addition of ammonium phosphate and oxygen allowed the depletion of sugars and the increase of the dilution rate and ethanol production (i.e. derived in higher ethanol productivities). (3) Hernández-Cortés et al. (2010) carried out continuous fermentations at laboratory scale, using two *S. cerevisiae* strains, feeding sterilized or non-sterilized agave juice and controlling or not controlling the pH during the cultures. These authors reported that no significant differences were found on the fermentative capability of *S. cerevisiae* S1 cultured at pH of 2.5 ± 0.5 (with no control of pH), between sterilized and non-sterilized media. Continuous agave juice fermentations showed higher productivities compared to batch fermentations; however, the maximum dilution rate reached in continuous fermentations (0.08 h^−1^) was considerably lower than the maximum *S. cerevisiae* specific growth rate calculated in batch systems (0.28 h^−1^). This fact suggested a nutritional limitation for *S. cerevisiae* in continuous culture fed with agave juice.

Agave juice has a low content of nitrogen, where the amino-acids are the main nitrogen (N) source. Valle-Rodríguez et al. (2012) and Díaz-Montaño et al. reported that the concentration of amino-acids in agave juice is up to 7000 times lower than their concentration found in grape juice (the average nitrogen content of agave and grape juices are 0.02 and 140 mg N L^−1^, respectively). In the tequila industry, the nitrogen deficiency is compensated by the addition of inorganic N-sources, such as ammonium sulfate or ammonium phosphate. However, agave juice could be not only deficient in N-source, requiring the supplementation of yeast growth factors (such as vitamins and amino-acids). This nutrimental limitation could be the origin of the slowness of fermentative capacity of yeast cultured in continuous, hindering a decrease in specific growth rate that operationally translates in a much lower dilution rate in the continuous bioreactor.

Due to its high level of protein, vitamin B complex and minerals, yeast extract might be used as a N-source and growth factor in yeast cultures, alleviating the nutritional limitation of agave juice (Ferreira et al. 2010) and enabling reaching higher dilution rates. In this scientific research, the effect of supplementing agave juice with yeast extract on the fermentative and aroma capabilities of a *S. cerevisiae* wild strain cultured in continuous was studied.

## Materials and methods

### Yeast strain

*Saccharomyces cerevisiae* strain S1 (culture collection number: CECT 13038 in WDCM412) was previously isolated from spontaneous agave juice fermentation and selected based on its fermentative capacity (Díaz-Montaño et al. 2008). The selected strain was stored at −70 °C in a 1:1 mixture of the propagation medium and a 50 % glycerol solution.

### Culture media

*Agave tequilana* Weber var. azul juice (agave juice) was collected from a local distillery (Compañía Tequilera La Quemada S.A. de C.V., El Arenal, Mexico) and stored at −20 °C. Agave juice employed in this work was derived from the same harvest at agave cultivation lands in the region Valles, Jalisco Mexico. It was conserved frozen in a big chamber at −20 °C and subsequently defrosted and filtered. Fermentation and propagation media consisted of agave juice adjusted with distilled water to 100 and 30 g L^−1^ of reducing sugars, respectively. Ammonium sulfate (1 g L^−1^, MP Biomedicals, Aurora OH, USA) and ammonium phosphate monobasic (4 g L^−1^, Productos Químicos Monterrey, Monterrey, Mexico) were added to both media. Yeast extract (4 g L^−1^, with a nitrogen content of 440 mg L^−1^; Becton, Dickinson and Company, Cuautitlán Izcalli, Mexico) was also supplemented to the fermentation medium. The nitrogen requirement of this yeast strain was previously investigated (Morán-Marroquín et al. 2008). Media were sterilized by autoclaving at 121 °C during 15 min.

### Inoculum preparation

The stored yeast strain was grown at 30 °C and 250 rpm in 100 mL propagation medium contained in a previously sterilized 250 mL Erlenmeyer flask. After 24 h of incubation, 10 mL of fermented medium were inoculated to 100 mL of fresh propagation medium contained in a 250 mL Erlenmeyer flask, which was cultured for 12 h at the same conditions. Yeast population and viability were estimated using a Neubauer counting chamber and methylene blue staining (Smart et al. 1999).

### Continuous fermentation conditions

Continuous fermentations were performed in a 3 L bioreactor (Applikon^®^ Biotechnology, Delft, The Netherlands) with an operation volume of 1.5 L, at 30 °C and 250 rpm. Before switching to continuous mode, sterilized fermentation medium was inoculated with 3.5 × 10^6^ cells mL^−1^ and initiated on batch mode with no aeration for 14 h. Sterile or non-sterile fermentation medium was fed using two peristaltic pumps (Cole-Parmer Company, Barrington, IL, USA) accurately synchronized to feed fresh medium and to extract fermented medium. The starting flow rate of feeding medium was 1 mL min^−1^ (dilution rate, *D* = 0.04 h^−1^). Air was injected into the bioreactor at a volumetric flow of 0.02 vvm, through a sterile PolyVENT™ filter (Whatman™ International Ltd, Maidstone, UK). Five residence times were set after a fermentation condition was modified to reach a new stationary state. Samples were taken every 12 h for the first three residence times and then every 6 h for each experimental condition.

### Analytical methods

Biomass was determined by dry weight. Five millilitres of fermented must were centrifuged at 5554×*g* for 15 min. Pellets were washed twice with 5 mL of distilled water and centrifuged at the above conditions; subsequently, pellets were dried at 50 °C during 24 h. Assays were made twice. The supernatants were stored at −20 °C for subsequent analysis.

Yeast population was estimated using a Neubauer counting chamber and its viability was determined by the methylene blue staining method (Smart et al. 1999). Concentration of reducing sugars, ammonium and ethanol were determined respectively by the Miller method (Miller 1959), the Chaney method (Chaney and Marbach 1962) and an enzymatic analyzer (YSI model 2700 Series Biochemistry Analyzer, Yellow Springs Instruments Inc., Yellow Springs, OH, USA) equipped with a Ethanol Membrane (No. 2786, Alcohol oxidase), Ethanol Buffer (No. 1579) and Ethanol standards (No. 2790: 2.00 and 3.20 g L^−1^). The ethanol is oxidized to hydrogen peroxide and acetaldehyde, and the hydrogen peroxide is detected amperometrically.

Fructose, glucose, glycerol, acetic acid, succinic acid, acetoin, diacetyl and ethanol concentrations were determined by a HPLC (Varian ProStar) coupled to a refractive index detector (Varian ProStar 355). Supernatants of the fermented samples were filtered with a 0.2 μm pore diameter filter and injected (20 µL) in an Animex HPX-87H Bio-Rad column, separation was performed using sulphuric acid 0.005 M as mobile phase at 0.4 mL min^−1^ and 40 °C. Quantification was based on five-point calibration curves for each analyzed compound: from 0.2 to 1 g L^−1^ for diacetyl, acetic and succinic acids, from 0.4 to 2 g L^−1^ for acetoin, from 1 to 5 g L^−1^ for glycerol and glucose and from 4 to 20 g L^−1^ for ethanol and fructose.

Minor volatile compounds in must were determined by headspace gas chromatography (GC) employing a Hewlett-Packard Head-space HP 7684E model (Hewlett Packard, Palo Alto, CA, USA) connected to a Hewlett-Packard 6890 Series gas chromatograph (Hewlett Packard). Detection was made using a flame ionization detector (FID) operated at 260 °C. The temperature of the column HP-Innowax (60 m × 320 µm × 0.25 µm) was initially maintained at 35 °C during 10 min; subsequently it was increased up to 210 °C at a rate of 3.5 °C min^−1^. Helium was used as carrier gas at 1.5 mL min^−1^, the flows of the auxiliary gases (nitrogen and hydrogen) and air were 30 and 300 mL min^−1^, respectively. Quantification was based on five-point calibration curves for each analyzed compound: from 0.2 to 1 mg L^−1^ for 2-butane, isoamyl acetate, ethyl decanoate, ethyl hexanoate, ethyl octanoate, furfuraldehyde and 2,3-butanodione; from 4 to 20 mg L^−1^ for 1-butanol, acetaldehyde, ethyl acetate; from 8 to 40 mg L^−1^ for propanol, isobutanol, and 2-phenyl ethanol; and from 60 to 300 mg L^−1^ for methanol and isoamyl alcohol.

### Data analysis

Yield coefficients of reducing sugars to biomass and ethanol (Y_x/s_ and Y_p/s_, respectively) were calculated as the means of biomass or ethanol concentrations divided by the consumed reducing sugars, during the steady state, reached for each experimental condition of the continuous cultures. Productivities of ethanol (P_p_) were calculated as the means of ethanol concentrations multiplied by the dilution rate, during the steady state.

Principal component analysis of the volatile compounds in fermented musts was applied. A multifactorial ANOVA was used for comparing the different factors studied.

Two sign tests with pairing of results by treatments and two Wilcoxon signed-rank tests with pairing of results by treatments were applied for analyzing the results from the sensorial evaluation through the affective test.

The statistical software Statgraphics Plus 4 (Manugistics Inc., Rockville, USA) was used to process the data.

### Analysis of distilled fermented must

Fermented musts were double distilled in a 5 L cylindrical stainless steel vessel which was equipped with a copper heating coil. Identification and quantification of volatile compounds of distilled must (acetaldehyde, methanol, butanol, propanol, isobutanol, isoamyl alcohol, ethyl lactate and ethyl acetate), were determined by gas chromatography (Agilent Technologies chromatograph, HP 6890 N, Hewlett Packard) coupled to a flame ionization detector. The volume of injection was 0.5 µL and a HP/FFAP column (50 m × 2 µm × 0.30 µm) was operated on Split mode. Temperatures for the injector and detector were 220 °C and 240 °C, respectively. The column temperature was increased from 40 to 55, 55 to 165 and 165 to 220 °C, at 2.3, 10 and 30 °C min^−1^, respectively. Quantification was based on five-point calibration curves for each analyzed compound.

### Sensory analysis (affective and effective) of tequilas

Sensory analysis was performed through an affective (hedonic) test first and then an effective test by free choice qualitative description. These tests were performed by 50 consumers familiarized with the tequila beverage and residing in the main and oldest tequila producing region “Valles” in Jalisco, Mexico; in order to compare the distillate prepared in this work with two commercial tequilas (Blanco 100 % de agave): tequila Herradura (Grupo Industrial Herradura, Amatitán, Mexico) and tequila 4 Copas (Compañía Tequilera La Quemada S.A. de C.V., El Arenal, Mexico). Results from the affective test were statistically analyzed using two sign tests and two Wilcoxon signed-rank tests. The preference ranking of each tequila was calculated as the sum of triplicating the number of votes from consumers as “good”, duplicating “regular” votes and single “bad” votes with the following equation:Preference ranking = 3 × “good votes” + 2 × “regular votes” + 1 × “bad votes”.

The identity of each one of the three tequilas was disguised under the labels “A”, “B” and “C”, in order to avoid a brand/origin influence in their judgments. The free choice qualitative description consisted on each taster making his/her own list of attributes by free choice, according to their perception of each one of the three tequilas they tasted (same tequilas as in the affective test were employed in this test). The attributes are presented in Fig. 3.

## Results

### Effect of the supplementation of agave juice with yeast extract, on the compounds and kinetic parameters quantified during the continuous culture of *S. cerevisiae* S1

For all continuous fermentations, media were prepared by addition of 1 g L^−1^ ammonium sulfate and 4 g L^−1^ ammonium phosphate monobasic to agave juice (containing 100 g L^−1^ of reducing sugars). Furthermore, pH was not controlled during the cultures, since Hernández-Cortés et al. (2010) demonstrated that controlled pH did not have a significant effect on the fermentative capability of *S. cerevisiae* S1 strain. Uncontrolled pH fermentations naturally reached acid values (pH of 2.6 to 3.0). Taking this into account, the effects of the supplementation of yeast extract (at 4 g L^−1^) and air (at 0.02 vvm), and the medium sterilization on the fermentative capability of *S. cerevisiae* and productivity of continuous fermentation of agave juice were studied. Media were supplemented or not supplemented with yeast extract and subsequently, they were sterilized or non-sterilized. Additionally, continuous cultures were aerated (at 0.02 vvm) or not aerated (Table 1).

**Table 1:** Concentration of compounds and calculated parameters on the steady state of aerated (at 0.02 vvm) or non-aerated *S. cerevisiae* continuous cultures, fed with sterilized (SM) or non-sterilized medium (NSM)

Compound (g L^−1^)	Aeration and dilution rates (*D*)			
	0 vvm and 0.08 h^−1^		0.02 vvm and 0.08 h^−1^	
	SM	NSM	SM	NSM
*A* (*Non-supplemented with yeast extract*)				
Biomass	5.90 ± 0.21	4.93 ± 0.42	7.33 ± 0.29	6.83 ± 0.72
Ethanol	38.25 ± 0.84	37.48 ± 1.61	41.87 ± 0.62	39.97 ± 0.04
R sugars	14.46 ± 0.52	26.52 ± 0.33	4.73 ± 0.57	12.18 ± 1.63
Fructose	4.27 ± 1.23	26.27 ± 3.35	9.56 ± 2.33	10.76 ± 1.16
Glucose	2.17 ± 0.37	0.90 ± 0.14	2.62 ± 0.10	0.39 ± 0.01
Succinic acid	0.16 ± 0.01	0.06 ± 0.08	0.21 ± 0.09	0.12 ± 0.00
Glycerol	4.48 ± 0.38	3.08 ± 0.23	7.00 ± 0.11	4.44 ± 0.14
Diacetyl	nd	nd	nd	nd
Acetic acid	nd	nd	nd	nd
Acetoin	0.33 ± 0.12	nd	nd	0.18 ± 0.03
NH_4_^+^	1.80 ± 0.22	1.55 ± 0.17	1.17 ± 0.09	0.89 ± 0.05
Y_X/S_	0.069 ± 0.002	0.067 ± 0.005	0.077 ± 0.003	0.078 ± 0.007
Y_P/S_	0.447 ± 0.007	0.510 ± 0.020	0.439 ± 0.004	0.455 ± 0.008
P_P_	3.060 ± 0.067	2.998 ± 0.129	3.350 ± 0.050	3.198 ± 0.003
pH	3.0 ± 0.1	2.9 ± 0.3	2.8 ± 0.1	2.7 ± 0.3

Compound (g L^−1^)	Aeration and dilution rates (*D*)			
	0 vvm and 0.10 h^−1^		0.02 vvm and 0.12 h^−1^	
	SM	NSM	SM	NSM
*B* (*Supplemented with yeast extract*)^a^				
Biomass	6.57 ± 0.68	6.42 ± 0.23	8.16 ± 0.70	8.28 ± 0.90
Ethanol	42.95 ± 2.86	46.22 ± 3.20	45.11 ± 0.49	46.92 ± 1.87
R sugars	5.86 ± 0.48	6.60 ± 0.25	7.53 ± 0.89	7.23 ± 0.09
Fructose	3.56 ± 0.19	5.03 ± 0.32	2.91 ± 0.55	6.63 ± 0.04
Glucose	0.06 ± 0.04	0.41 ± 0.19	nd	0.57 ± 0.00
Succinic acid	0.08 ± 0.06	0.14 ± 0.02	0.08 ± 0.06	0.12 ± 0.04
Glycerol	2.76 ± 0.17	3.93 ± 0.17	3.00 ± 0.08	3.14 ± 0.06
Diacetyl	nd	nd	nd	nd
Acetic acid	nd	nd	nd	nd
Acetoin	nd	nd	nd	nd
NH_4_^+^	1.16 ± 0.12	1.59 ± 0.51	0.94 ± 0.05	0.77 ± 0.13
Y_X/S_	0.070 ± 0.007	0.069 ± 0.002	0.088 ± 0.007	0.089 ± 0.010
Y_P/S_	0.456 ± 0.028	0.495 ± 0.033	0.488 ± 0.001	0.506 ± 0.020
P_P_	4.295 ± 0.286	4.622 ± 0.320	5.413 ± 0.059	5.630 ± 0.224
pH	2.8 ± 0.3	2.7 ± 0.1	2.6 ± 0.2	2.6 ± 0.3

Medium contained agave juice (adjusted at 100 g L^−1^ of R sugars), ammonium sulfate (1 g L^−1^) and ammonium phosphate monobasic (4 g L^−1^). Y_X/S_ is biomass yield (g g^−1^). Y_P/S_ is ethanol yield (g g^−1^). P_P_ is ethanol productivity [g (Lh)^−1^]. Values are the means ± standard deviations (*n* = 3)

*R* sugars are Reducing sugars, *nd* compound not detected under the analysis conditions

^a^Experiments using media supplemented with yeast extract were performed at higher *D*, because this supplementation allowed this increase without losing stability in the continuous culture

For cultures non supplemented with yeast extract, aeration (at 0.02 vvm) showed significant differences (*P* > 0.05) for concentrations of biomass, residual reducing sugars, fructose, succinic acid and glycerol, compared to a non-aerated culture; while medium sterilization showed significant differences (*P* > 0.05) for concentrations of reducing sugars, fructose and succinic acid, compared to a non-sterilized medium (Table 1A). Aeration (at 0.02 vvm) promoted an increase of the kinetic parameters of *S. cerevisieae* S1 on continuous fermentations (Table 1A). In fact, aeration of fermentations fed with non-sterilized medium and non-supplemented with yeast extract, improved ethanol production from 37.5 to 40 g L^−1^, reducing sugars consumption from 73.5 to 88 g L^−1^ and ethanol productivity from 3.0 to 3.2 g (Lh)^−1^, for non-aerated and aerated (at 0.02 vvm) cultures, respectively.

For cultures supplemented with yeast extract and air (at 0 and 0.02 vvm) showed significant differences (*P* > 0.05) only for biomass concentrations; while the feeding of sterilized or non-sterilized medium to continuous fermentations did not show significant differences (*P* > 0.05) (Table 1B). It is worth noting that experiments using media supplemented with yeast extract (in Table 1B), were performed at higher dilution rate, because this supplementation allowed the increase from 0.08 to 0.12 h^−1^ without losing stability in the continuous cultures.

Concentration of ethanol, biomass and reducing sugars, and kinetic parameters of continuous cultures were also compared under the following conditions: (1) feeding non-supplemented media with yeast extract or air, and non-sterilized media (Table 1A); and (2) feeding supplemented media with yeast extract and air, and non-sterilized media (Table 1B). The ethanol and biomass productions and residual reducing sugars, as well as ethanol yield and productivity increased from 37.48 to 46.92 g L^−1^, from 4.93 to 8.28 g L^−1^, from 26.52 to 7.23 g L^−1^, from 0.447 to 0.506 g g^−1^ and from 2.998 to 5.630 g (Lh)^−1^, respectively for (1) and (2) conditions. Remarkably, the ethanol productivity increased by 88 %.

Concentrations of aroma compounds were also compared among continuous cultures supplemented or not with yeast extract and air, using either sterilized (SM) or non-sterilized (NSM) media (Table 2).

**Table 2:** Concentration of volatile compounds on the steady state of aerated (at 0.02 vvm) and non-aerated *S. cerevisiae* S1 continuous cultures fed with sterilized (SM) and non-sterilized medium (NSM)

Volatile compound (mg L^−1^)	Aeration and dilution rates			
	0 vvm and 0.08 h^−1^		0.02 vvm and 0.08 h^−1^	
	SM	NSM	SM	NSM
*A* (*Non-supplemented with yeast extract*)				
Acetaldehyde	50.52 ± 0.39	46.26 ± 2.12	39.55 ± 0.65	46.02 ± 0.32
Methanol	151.08 ± 6.46	128.26 ± 0.04	176.54 ± 23.23	111.88 ± 0.91
Propanol	51.16 ± 0.08	44.25 ± 0.08	92.59 ± 13.99	50.22 ± 1.90
Butanol	1.96 ± 0.00	1.03 ± 0.05	2.32 ± 0.32	0.95 ± 0.09
Isobutanol	10.15 ± 0.37	13.98 ± 0.31	18.31 ± 2.63	15.50 ± 0.57
Isoamyl Acohol	29.39 ± 0.63	23.71 ± 0.31	24.94 ± 6.04	17.93 ± 0.68
2-Phenylethanol	7.74 ± 0.52	8.66 ± 0.67	7.79 ± 1.20	13.46 ± 0.17
Ethyl acetate	17.53 ± 3.25	12.89 ± 0.25	5.89 ± 0.34	14.90 ± 0.73
Isoamyl acetate	0.39 ± 0.29	0.24 ± 0.04	0.09 ± 0.02	0.16 ± 0.00
Ethyl hexanoate	0.18 ± 0.26	0.11 ± 0.01	0.04 ± 0.01	0.10 ± 0.00
Ethyl octanoate	0.18 ± 0.11	0.06 ± 0.02	0.09 ± 0.03	0.03 ± 0.02
Ethyl decanoate	nd	nd	0.20 ± 0.07	nd
Phenethyl acetate	0.06 ± 0.01	0.16 ± 0.04	0.09 ± 0.02	nd
α-terpineol	0.21 ± 0.17	0.11 ± 0.06	0.61 ± 0.14	0.07 ± 0.09
2, 3 Butanedione	0.60 ± 0.13	1.94 ± 0.25	3.12 ± 0.54	0.29 ± 0.03

Volatile compound (mg L^−1^)	Aeration and dilution rates			
	0 vvm and 0.10 h^−1^		0.02 vvm and 0.12 h^−1^	
	SM	NSM	SM	NSM
*B* (*Supplemented with yeast extract*)				
Acetaldehyde	57.47 ± 3.12	30.33 ± 3.93	59.51 ± 0.35	30.45 ± 2.03
Methanol	103.04 ± 10.05	134.77 ± 3.13	100.16 ± 9.56	125.03 ± 0.91
Propanol	69.94 ± 5.88	65.93 ± 4.68	65.74 ± 4.48	70.02 ± 0.99
Butanol	1.30 ± 0.36	0.73 ± 0.58	0.55 ± 0.78	0.97 ± 0.01
Isobutanol	16.39 ± 0.86	16.81 ± 0.73	18.41 ± 4.37	15.23 ± 0.10
Isoamyl Acohol	53.74 ± 0.76	58.52 ± 3.57	51.88 ± 4.79	49.14 ± 0.74
2-Phenylethanol	10.44 ± 1.24	4.82 ± 1.94	5.71 ± 2.87	3.91 ± 0.74
Ethyl acetate	14.73 ± 0.44	46.29 ± 2.56	16.09 ± 0.42	18.15 ± 0.98
Isoamyl acetate	0.91 ± 0.49	3.10 ± 0.08	1.08 ± 0.51	0.88 ± 0.04
Ethyl hexanoate	0.38 ± 0.18	0.84 ± 0.07	0.34 ± 0.15	0.40 ± 0.01
Ethyl octanoate	0.14 ± 0.08	0.11 ± 0.16	0.07 ± 0.09	0.05 ± 0.06
Ethyl decanoate	0.03 ± 0.04	0.06 ± 0.08	0.01 ± 0.01	nd
Phenethyl acetate	0.26 ± 0.01	0.16 ± 0.09	0.18 ± 0.26	nd
α-terpineol	0.08 ± 0.02	nd	nd	nd
2, 3 Butanedione	0.76 ± 0.50	1.67 ± 0.69	0.30 ± 0.42	0.99 ± 0.04

*nd* Compound not detected under the analysis conditions. Values are the means ± standard deviations (*n* = 3)

An analysis of principal components revealed the effect of yeast extract addition on the production of minor volatile metabolites, obtaining four principal components (PC), which explained 82.05 % of the total variation of the experimental data. Subsequently, an analysis of variance was applied to those principal components revealing that supplementation of yeast extract, air and medium sterilization had significant effects on PC-1, PC-2 and PC-3, respectively (Table 3).

**Table 3:** Analysis of variance (ANOVA) of principal components (PC) obtained from volatile compounds of aerated (at 0.02 vvm) or non-aerated *S. cerevisiae* continuous cultures fed with sterilized (SM) or non-sterilized (NSM) agave juice supplemented or not supplemented with yeast extract

	PC-1	PC-2	PC-3	PC-4
Explained variance (%)	33.46	19.79	15.41	13.38
Yeast extract (*p* value)	0.0000	0.3761	0.7171	0.1027
Aeration rate (*p* value)	0.4194	0.0081	0.0740	0.7873
Fermentation medium (*p* value)	0.0910	0.2911	0.0363	0.0673

**Fig. 1:**
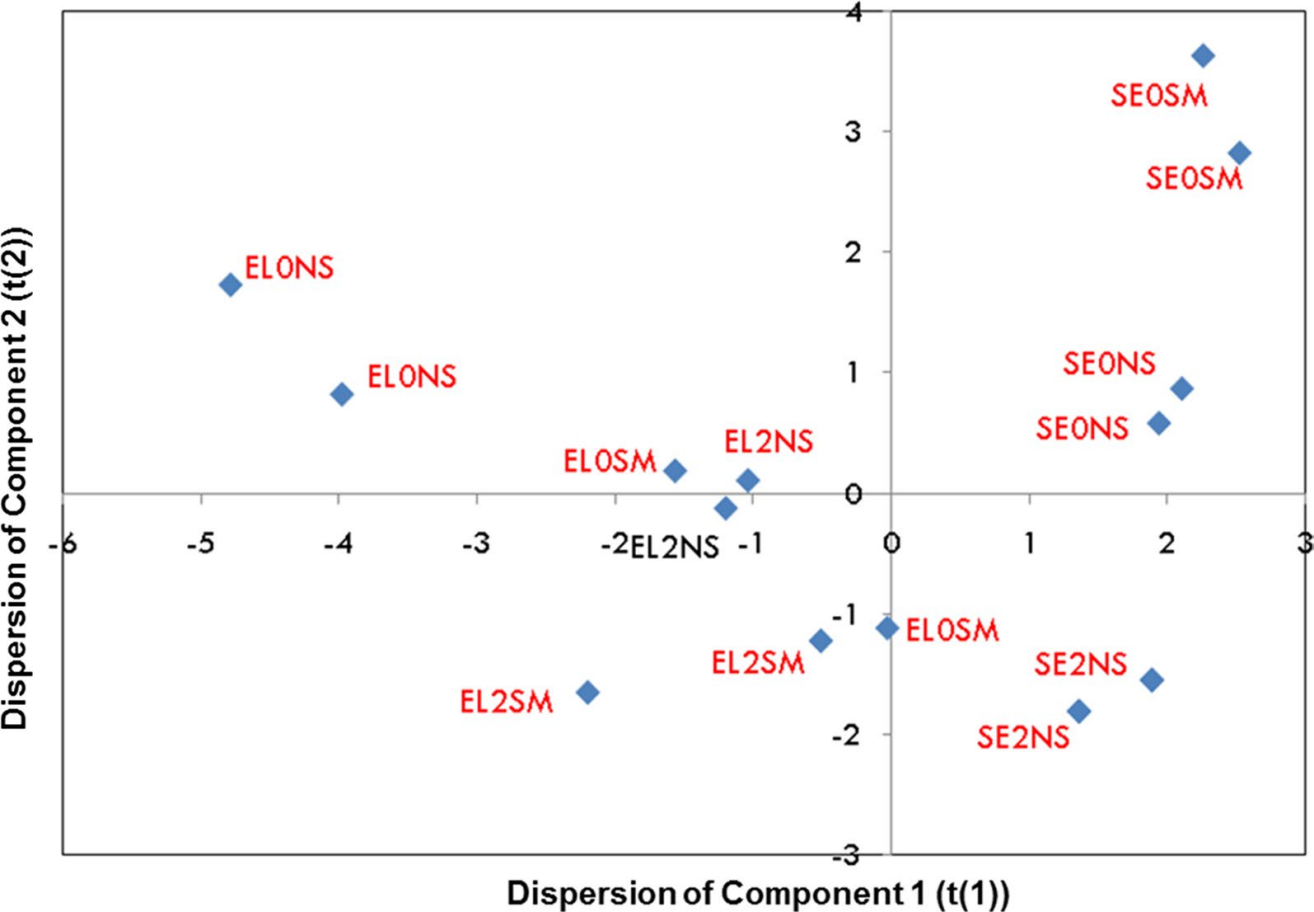
Diagram of dispersion of principal components PC-1 and PC-2, obtained from the concentration of aromatic compounds on the steady state of 0.02 vvm aerated (2) and non-aerated (0) *S. cerevisiae* continuous cultures fed with sterilized (SM) and non-sterilized (NS) media, supplemented (EL) or not (SE) with yeast extract

PC-1 differentiated volatile compound profiles in cultures with and without addition of yeast extract. Fermentations showed negative scores with yeast extract and positive scores without yeast extract for PC-1 (Fig. 1). Moreover, PC-2 differentiated those cultures aerated from those non-aerated. Aerated cultures showed negative scores and non-aerated cultures positive scores for PC-2 (Fig. 1).

### Concentration of regulated compounds in distillates of fermented must (tequila)

**Fig. 2:**
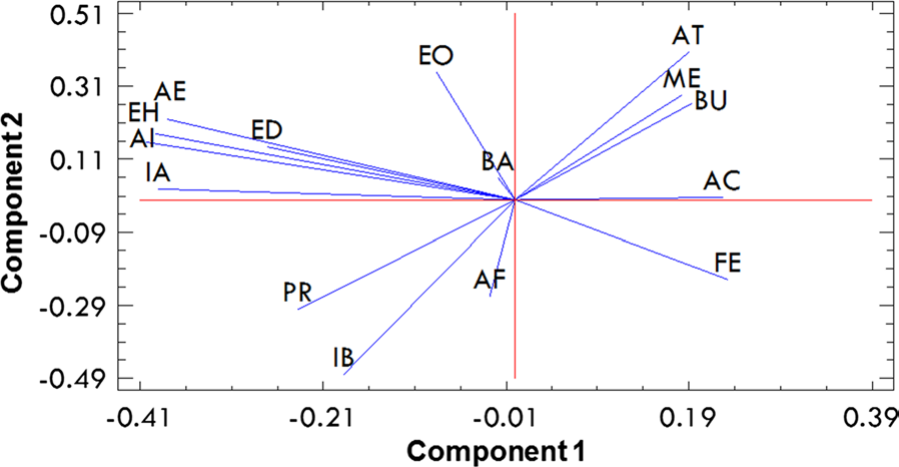
Principal component (PC-1 and PC-2) weights of the concentration of aromatic compounds on the steady state of *S. cerevisiae* continuous cultures supplemented with yeast extract. *AC* Acetaldehyde, *AE* Ethyl acetate, *ME* Methanol, *BA* 2,3-butanodione, *PR* Propanol, *IB* Isobutanol, *AI* Isoamyl acetate, *BU* Butanol, *IA* Isoamyl alcohol, *EH* Ethyl hexanoate, *EO* Ethyl octanoate, *ED* Ethyl decanoate, *AT* Alpha-terpineol, *AF* Phenyl acetate, *FE* 2-phenylethanol

Fermented musts, obtained during the steady state of continuous fermentations supplemented with yeast extract, were double distillated using the procedures commonly utilized in the tequila industry (Cedeño 2003; NOM-006-SCFI-2012 2012). The concentration of compounds in the distillates were determined and compared to those regulated by the Official Mexican Norm NOM-006-SCFI-2012 (2012) (Table 4). Differences on each of the main aroma compounds are presented in Fig. 2. The compounds that varied the most were esters (PC-1 < 0.35 and PC-2 > 0), followed by alcohols (PC-1 > 0.15 and PC-2 > 0.25). The production of the ketone 2,3-butanodione did not vary significantly. Moreover, as it is shown in Table 4, aeration and sterilization of the fermentation medium did not show influence on the concentration of the volatile compounds in the distillates.

**Table 4:** Concentration of volatile compounds assayed in distillates obtained from fermented musts of aerated (at 0.02 vvm) and non-aerated *S. cerevisiae* continuous cultures fed with sterilized (SM) and non-sterilized (NSM) agave juice supplemented with yeast extract

Compound	Dilution and aeration rates				
(mg per 100 mL of	0.10 h^−1^ and 0 vvm		0.12 h^−1^ and 0.02 vvm		NOM^a^
alcohol)	SM	NSM	SM	NSM	
Acetaldehyde	17.42 ± 0.96	12.82 ± 0.98	11.13 ± 0.91	25.57 ± 1.35	0–40
Methanol	180.15 ± 11.33	227.43 ± 19.81	252.67 ± 23.84	200.32 ± 11.94	30–300
*Fusel alcohols*	280.58 ± 16.69	258.08 ± 20.67	269.91 ± 24.16	349.49 ± 19.58	20–500
2-butanol	137.68 ± 8.09	125.69 ± 10.22	151.11 ± 13.32	173.92 ± 9.68	
1-propanol	nd	nd	nd	nd	
Isobutanol	34.43 ± 1.50	27.26 ± 1.46	23.89 ± 1.38	40.45 ± 1.48	
1-butanol	1.87 ± 0.52	nd	2.06 ± 0.86	2.54 ± 0.67	
Isoamyl and amyl alcohols	106.61 ± 6.58	105.13 ± 8.99	92.86 ± 8.60	132.58 ± 7.75	
*Esters*	8.66 ± 0.25	11.35 ± 0.64	5.45 ± 0.23	9.71 ± 0.55	2–200
Ethyl lactate	nd	nd	2.14 ± 0.15	1.23 ± 0.09	
Ethyl acetate	8.66 ± 0.25	11.35 ± 0.63	3.31 ± 0.09	8.48 ± 0.46	
*Furfurals*	2.98 ± 0.21	nd	nd	nd	0–4

nd Compound not detected under the analysis conditions

Values are the means ± standard deviations (*n* = 3)

^a^NOM: official mexican norm of alcoholic beverages-tequila specifications (NOM-006-SCFI-2012 2012). Ranges of minimum and maximum allowed concentrations in tequila

### Sensory analysis of the tequila prepared in this work compared to two commercial tequilas

#### Affective test

Fifty consumers participated in the preference study, first affectively qualifying the tested tequilas (the tequila prepared in this work, tequila Herradura and tequila 4 Copas) with a hedonic test as “good”, “regular” or “bad”, according to their perceived taste for each tequila (Table 5). The tequila prepared in this work received a preference ranking of 95 points, while tequila Herradura and tequila 4 Copas received 101 and 92 points, respectively. By performing two statistical sign tests with pairing of results by treatments and two Wilcoxon signed-rank tests with pairing of results by treatments (for confirming), no significant differences were found between the tequila prepared in this work and the commercial tequila Herradura (*p* = 0.7656 and 0.4451 for sign and Wilcoxon tests, respectively) nor between the tequila from this work and the commercial tequila 4 Copas (*p* = 0.8875 and 0.6450 for sign and Wilcoxon tests, respectively).

**Table 5:** Preference rankings calculated from the votes of fifty consumers, according to the perceived taste for each tested tequila as “good”, “regular” or “bad”

Tequila	Qualification given by the consumer			Preference ranking
	Good	Regular	Bad	
Prepared in this work	14	17	19	95
Herradura	15	21	14	101
4 Copas	14	14	22	92

“Herradura” and “4 copas” are well accepted commercial tequilas. The preference ranking of each tequila was calculated as the sum of triplicating the number of votes from consumers as “good”, duplicating “regular” votes and single “bad” votes with the following equation:Preference ranking = 3 × “good votes” + 2 × “regular votes” + 1 × “bad votes”

#### Effective test

**Fig. 3:**
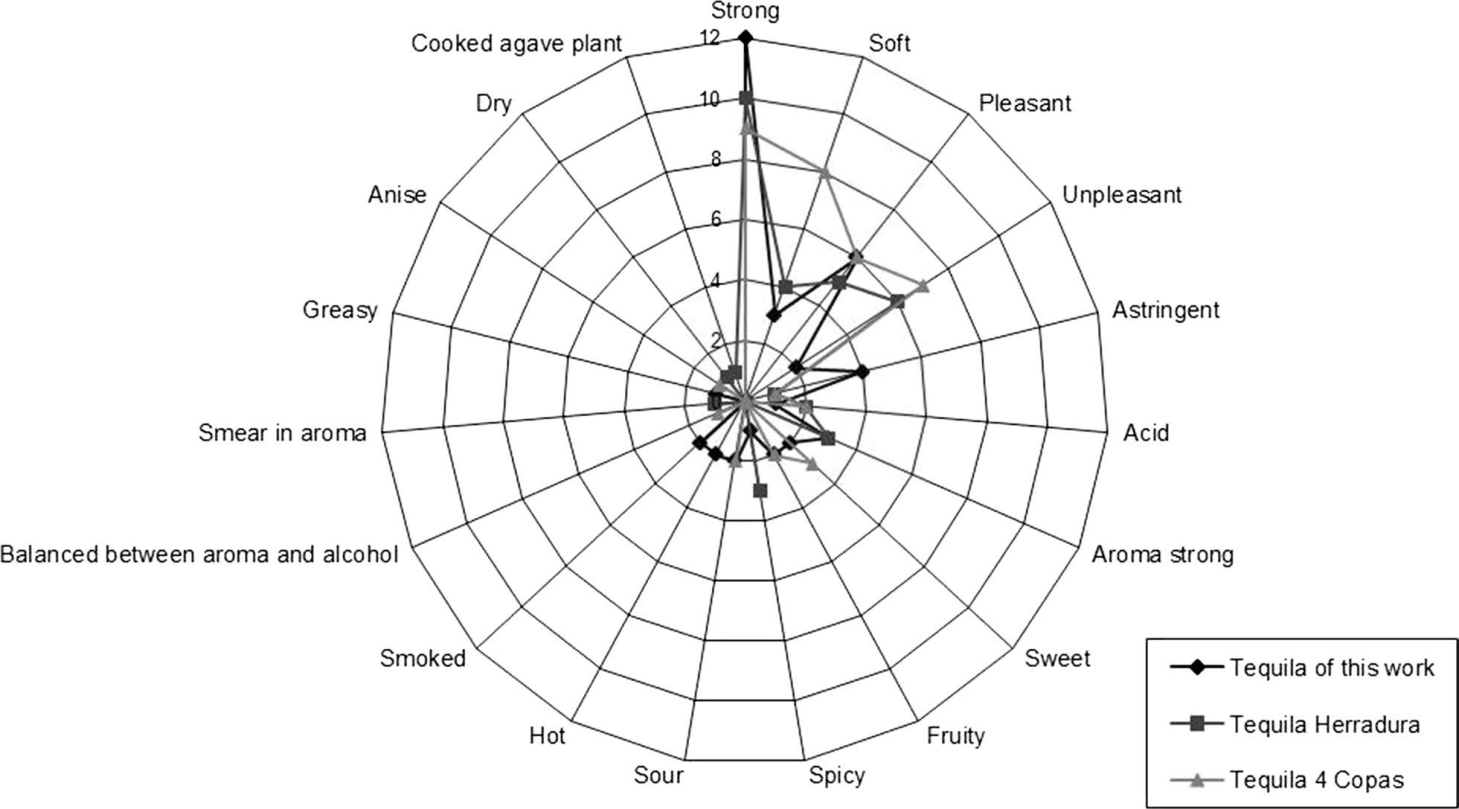
Webplot of the free choice qualitative description (as effective test) of the tequila produced in this study and two commercial tequilas (tequila Herradura and tequila 4 Copas). Based on evaluations given by 50 consumers

The free choice qualitative description showed that the attribute or adjective that summarizes the judgement of the consumers, from their experience of the tequilas they tasted, is if they found it either pleasant or not (Fig. 3). Six evaluators described the tequila of this study pleasant, while only two of them found it unpleasant to their taste. This represented only 33 % of the population that liked it. While surprisingly for the two commercial tequilas, 6 evaluators perceived tequila 4 Copas unpleasant, while 5 of them found it pleasant; and 7 of them found tequila Herradura pleasant while 6 consumers did not like it. This sensory evaluation with assignation of descriptors to the tequilas, favored the tequila of this work above commercial tequilas.

As fashion in the survey shows, the evaluators found the tequila produced in this work relatively stronger (12 votes) when compared to the commercial tequilas: Herradura (10) and 4 Copas (9). Matching with this description of them, the tequila that was found the softest was 4 Copas (8 votes), followed by Herradura (4) and finally the least number of testers thought that this study tequila was soft (3).

As for descriptors in detail regarding taste and aroma, it is remarkable to mention that four consumers found the tequila of this study astringent, while only one of them described each of the commercial tequilas astringent. Three evaluators found tequila Herradura strong in aroma. Three of them as well, perceived tequila 4 Copas as sweet, while two of them found the tequila of this study also sweet. Related to this last descriptor, two of the testers encountered the tequila of this work and 4 Copas as fruity. Three consumers described tequila Herradura as spicy, while only one of them stated our tequila spicy and none of them assigned this descriptor to tequila Herradura.

Regarding other descriptors, they were not perceived in high number by the evaluators. Only two of them described the commercial tequilas as acid, other two of them found our tequila and tequila 4 Copas sour, other two testers perceived our tequila as hot and other two ones described it as rather smoked.

## Discussion

Agave juice is obtained by extraction of cooked heads of *Agave tequilana* Weber var. azul and used to be fermented in the tequila industry; however, it is nutritionally deficient to support a balanced yeast growth, resulting in low fermentative rates and high residual sugars, which severely limit the implementation of yeast continuous fermentations. In order to alleviate nutritional deficiencies (mainly growth factors), the approach in this work was supplementing the agave juice with yeast extract (also ammonium was initially added as a nitrogen source), during the *S. cerevisiae* S1 continuous fermentations. Additionally, continuous fermentations were aerated or not aerated, and fed with sterilized and non-sterilized media. Micro-aeration was considered as an important condition for the fermentation process, since yeasts require low amounts of oxygen for synthesizing some essential lipids to assure cell membrane integrity (Mauricio et al. 1997). The absence of media sterilization was considered in this work in order to simplify the process and reduce the costs of tequila production.

An important parameter, limiting the growth of other microorganisms different to the inoculated yeast, was the acid pH (from 2.6 to 3) prevailing in all cultures. On the other hand, microscopic observations were carried out during the continuous cultures and no microbial contamination was detected. A further aspect, indicating the absence of microbial contamination was the high ethanol concentrations, yields (near to the theorical maximum) and productivities reached in this work, feeding non-sterilized media, which were even higher than feeding sterilized media (Table 1).

Higher dilution rates (*D*) and fermentative capacity of *S. cerevisiae* were attained by supplementing the medium with yeast extract. With this supplementation, *D* was increased from 0.08 to 0.12 h^−1^. Indeed, for continuous cultures non supplemented with yeast extract, a maximum dilution rate (*D*) of 0.08 h^−1^ was attained; while for cultures supplemented with yeast extract, non-aerated and aerated, *D* of 0.10 and 0.12 h^−1^ were reached, respectively; since at these *D*, reducing sugars were depleted and ethanol productions were maximal. Higher *D* showed lower reducing sugars consumption and ethanol production, and instability of the system; while lower dilution rates did not show significant differences regarding the selected dilution rates (data not shown).

Micro-aeration (at 0.02 vvm) of continuous fermentations non-supplemented with yeast extract, promoted an increase of biomass and ethanol concentrations and a decrease of residual reducing sugars concentration (Table 1A); while micro-aeration of continuous fermentations supplemented with yeast extract, promoted only an increase on biomass.

Ethanol and biomass productions, reducing sugars consumption, and ethanol yield and productivity of *S. cerevisiae* S1 continuous fermentations were noticeably improved by supplementation of yeast extract and air (Table 1).

Low growth and ethanol production rates of yeast are often associated with deficiency of nutrients in natural media, such as plant extracts or fruit juices (Bisson 1999; Díaz-Montaño et al. 2008). Hence, supplementation of agave juice with yeast extract stimulated higher *S. cerevisiae* fermentative efficiency, since yeast extract contains growth factors, including lipids (i.e. ergosterol and sterols), which might confer higher robustness and integrity to the yeast cellular membrane, allowing a superior tolerance to ethanol (Visser et al. 1990).

Feeding non-sterilized or sterilized medium did not show significant differences (*P* > 0.05), regarding the kinetic parameters (Table 1 B). It is worth noting that microbial contamination was not observed even when non-sterilized medium was fed. Acid pH values (from 2.6 to 3) found during the cultures (Table 1), might have limited microbial contamination. This low pH condition suggested a reduced probability of contamination for prolonged continuous cultures and explained why no significant differences were found between continuous cultures fed with sterilized or non-sterilized media. This finding could positively impact the economic balance of the industrial production of tequila.

Yeast extract is an inexpensive source of nutrients, since it could be recovered and recycled from the same fermented must; however, other considerations must be taken into account before proposing this component as a supplement in a commercial scale; particularly, an adequate profile of aroma compounds contained in the product must be guaranteed. In fact, yeast extract contains proteins and amino-acids which are precursors of fusel alcohols and volatile compounds, which could modify the beverage bouquet (Ehrlich 1907; Arrizon and Gschaedler 2007); Garde-Cerdán and Ancín-Azpilicueta 2007).

The results showed that no significant differences were found for concentrations of volatile compounds analyzed in continuous fermentations supplemented or not with yeast extract or air (Table 2). Furthermore, the volatile compound concentrations in the distillates (tequila), obtained from fermented musts of continuous cultures supplemented with yeast extract, were comprised between the allowed ranges established by the Mexican regulation of tequila (NOM-006-SCFI-2012 2012, official mexican norm of alcoholic beverages-tequila specifications) (NOM-006-SCFI-2012 2012); which suggested the feasibility to produce tequila, using continuous fermentations fed with agave juice supplemented with yeast extract (Table 4). On the other hand, aeration and sterilization of the fermentation medium did not influence on the concentration of the volatile compounds in the distillates (Table 4).

Methanol concentrations were found near to the maximum limit allowed by the Mexican norm (NOM-006-SCFI-2012 2012). It is worth noting that methanol is not synthesized in yeast metabolism, but it is produced by demethylation of agave juice pectins, released during the cooking of agave heads (Waleckx et al. 2008). In this work, a high methanol concentration was found in the feeding medium (up to 110 g L^−1^), which was similar to that concentration obtained in the fermented must (up to 134.77 g L^−1^).

It is worth noting that the profile and concentration of volatile compounds in the fermented must is often useful to predict the quality of the final product; however, they are not accurately related to the consumer preference (Jackson 2008). For this reason, the preference of the double-distillate, obtained from continuous fermentations of agave juice supplemented with yeast extract and air, and feeding sterilized medium, was compared with two commercial tequilas (4 Copas and Herradura), according to a sensory analysis through first an affective test and then an effective test. In the hedonic test, tequila 4 Copas and the tequila of this work received the major number of votes as a “bad” tequila (22 and 19 votes, respectively), while tequila Herradura received the most of votes (21) as a “regular” tequila. As the preference ranking indicated, the tasted tequilas were practically ranked as “regular”, since the theoretical ranking values were around of 100 points. Despite this, there were no statistical differences (sign tests and Wilcoxon signed-rank tests) found between the tequila of this work and any of the commercial tequilas. Results from the effective test, positioned the tequila of this work more pleasant to consumers (only two consumers found it unpleasant) when compared to the two commercial tequilas, while most of them also found our tequila strong, same result when the commercial tequilas were qualified. Most of the consumers assigned descriptors linked to basic degustation savours (acid, sweet, sour), besides common distillated alcoholic beverages descriptors such as astringency, aroma strength, fruitiness, spiciness, hotness and smoked-likeness.

The implementation of this technological alternative is feasible on an industrial scale. Work is ongoing to optimize yeast extract addition to agave juice, and a suitable fermenter is being designed for the continuous fermentation at pilot scale.

## Conclusions

Results indicated the possibility of overcoming agave juice nutritional limitations by the supplementation of yeast extract and air, reaching higher fermentative capabilities of *S. cerevisiae*. Continuous fermentations of agave juice supplemented with yeast extract and air increased ethanol productivity 1.88-fold compared to that productivity obtained in the fermentation not supplemented.

Remarkably, the low pH levels (from 2.6 to 3) observed during fermentations could reduce the probability of microbial contamination in prolonged continuous cultures. In fact, microbial contamination was not perceived during the cultures, even if the feeding media was not sterilized.

The yeast extract supplementation in the continuous cultures did not modify significantly the concentrations of volatile compounds in the fermented must (compared to a non-supplemented fermentation), showing the feasibility of adding yeast extract to agave juice for producing tequila.

Principal component analysis differentiated volatile metabolites from continuous fermentations supplemented or not with yeast extract.

Distillates obtained from fermented musts were under the allowed concentration ranges of volatile compounds for tequila regulations and they were well accepted by 50 evaluators that tasted them. The tequila of this work showed similar preference level when compared to that of two well-known commercial tequilas. Finally, the results from a sensorial analysis favoured the tequila prepared in this work.

## Abbreviations

PC: principal component weight from an ANOVA analysis
*D*: dilution rate (h^−1^)
P: productivity [g (Lh)^−1^]
